# The Emerging Role of Sigma Receptors in Pain Medicine

**DOI:** 10.7759/cureus.42626

**Published:** 2023-07-28

**Authors:** Joseph Pergolizzi Jr, Giustino Varrassi

**Affiliations:** 1 Operations, NEMA Research, Inc., Naples, USA; 2 Pain Medicine, Paolo Procacci Foundation, Rome, ITA

**Keywords:** cardiac disorders, neurodegenerative diseases, neuropathic pain, pain, sigma-receptors

## Abstract

Sigma receptors are protein chaperones with the unexpected characteristic of being activated by ligand binding. As such, they represent intriguing new targets for potential drug development. As a protein chaperone, these “receptors” escort proteins from the endoplasmic reticulum to their destinations and act to correct misfolded proteins. The two subtypes of sigma receptors, named σ1 and σ2, are markedly distinct from each other. Agonists and antagonists at these receptors show promise as new drug targets, addressing a range of diseases including neurodegenerative disorders, cancer, and cardiac disorders, and may also be analgesic agents and rehabilitation drugs for opioid use disorder. As an analgesic, sigma receptors seem to be more effective in treating neuropathic than nociceptive pain. New bifunctional compounds are being developed with opioids, because agents targeting sigma receptors may have an opioid-sparing effect. The pipeline of agents based on the sigma receptors is long and may treat things from Fragile X syndrome to Parkinson's disease to Huntington’s disease to cancer. A novel agent ADV502 acts as a high-affinity σ1 antagonist and partial agonist at the µ-opioid receptor and may be an important agent both for the treatment of neuropathic cancer pain and for rehabilitation of opioid use disorder. Since there has been little recent innovation in pain medicine regarding new compounds and drug targets, drugs that affect the sigma receptor system seem promising and encouraging.

## Introduction and background

In 1976, sigma receptors were thought to be a form of opioid receptor [1]. Our evolving understanding of sigma receptors has now expanded to recognize two types of ligand-biased protein chaperones, known as σ1 and σ2. They are found abundantly in the endoplasmic reticulum and migrate to chaperone properly folded proteins to their cellular destinations. Perhaps the most remarkable finding about these enigmatic “receptors” is that they have associations with a broad range of conditions, from neurodegenerative disorders to cancer, from amyolateral sclerosis to depression [2]. They represent exciting new targets for drug development, particularly for pain care. Drugs developed to bind with these receptors are termed agonists and antagonists and various combinations have been developed, including agents that include µ-opioid receptor agonists. The potential serviceability of these novel agents for pain control remains to be evaluated, but they may offer a nonopioid way to control pain without the adverse effects of opioid analgesic therapy. Particularly intriguing is that these drug targets hold promise in particular for the treatment of neuropathic pain syndromes or mixed pain with a neuropathic component.

This paper is based on three presentations given at a pain conference in Cancun, Mexico in April 2023. These presentations are narrative reviews as well as clinical experiences and academic insights of the authors on these topics.

## Review

Advances in σ-receptor research and drug development

Sigma receptors have emerged as a revitalized and extremely interesting topic of basic science research. The σ1 receptor was first cloned in 1996, but its crystal structure was not published until 20 years later in 2016 [3, 4]. In 2017, the gene associated with what came to be known as the σ2 receptor subtype was identified. It was cloned in 2017 and its crystal structure was published in 2021 [5, 6]. Both receptors are highly conserved in mammals, facilitating preclinical investigations, but the two receptors are not structurally similar to each other. At first thought to be a new type of opioid receptor, these receptors instead were chaperones whose primary function involved the proper folding of proteins. Since they were regulated by ligands, they served the role of a receptor although they were chaperones (see Table 1).

**Table 1:** Comparison of the σ1 and σ2 receptors [7]. GPCR, G protein-coupled receptor

Characteristic	σ1	σ2
223 aa (26 kD) protein with two transmembrane domains	Yes	No
176 aa (21 kD) protein with two transmembrane domains	No	Yes
Non-GPCR (nonopioid)	Yes	Yes
C-terminus has chaperone action	Yes	Yes
Chaperone helps proteins fold properly	Yes	Yes
Chaperone is regulated by ligands (“receptor” function)	Yes	Yes

Pre- or post-translation misfolding of proteins can cause loss or reduction of functionality, and aggregates of misfolded proteins can cause endoplasmic reticulum stress, associated with disease. If protein misfolding cannot be corrected, cells can die [8]. Chaperone proteins serve several purposes: they bind to properly folded proteins and deliver them to their proper destination; they can bind to misfolded proteins and resolve the misfolding. Misfolded proteins have an aberrant conformation and tend to aggregate together, playing a role in the progression of certain neurodegenerative conditions [9]. Unlike other types of protein chaperones, these σ-receptor proteins are sensitive to ligands. The σ-receptor agonists and antagonists that are being developed are being designed to serve as a type of pharmacologic chaperone [2].

The σ1 receptors have the ability to interact with µ-opioid receptors in ways that are now being elucidated. The σ1 receptors are expressed in certain areas of the body associated with pain control, and the ligands for σ1 do not alter normal sensory thresholds although they decrease the sensory gain that can occur with sensitization. Such sensory gain may occur as hyperalgesia or allodynia. Agents that act as σ1 receptor antagonists potentiate the antinociceptive activity of various µ-opioid receptor agonists. This suggests an intriguing possibility, namely that a dual or bifunctional analgesic agent may be created with a σ1 antagonist combined with a µ-opioid receptor agonist. Such a dual-action agent might confer analgesic benefits with fewer of the typical opioid-associated side effects [10]. Using computer-based models, bifunctional compounds have been considered and their specific features assessed [11]. A candidate drug (EST73502, Esteve Pharmaceuticals, Barcelona, Spain) was selected and evaluated in preclinical studies. It has entered clinical trials for the treatment of pain associated with osteoarthritis as the first dual µ-opioid receptor agonist/σ1 receptor antagonist [12, 13]. This drug might be considered the first-in-class bifunctional compound [10].

Endogenous opioids modulate both the sensation and perception of pain. This system for managing pain in the body may be superior to all others, so an agent working with this system has certain inherent advantages. The combination approach poses the theoretical advantage of providing better benefits with fewer opioid-related adverse effects. The drug candidate, now known as ADV-502 rather than EST73502, was demonstrated effective during in vivo preclinical studies for acute pain in the paw-pressure and tail-flick test [13]. ADV502 was likewise effective in animal studies in reducing visceral pain caused by intracolonic capsaicin [13]. This combination product demonstrated greater action and effectiveness in acute pain syndromes than might be expected from σ-receptor antagonist monotherapy. Visceral pain control with ADV502 suggests that the bifunctional candidate agent ADV502 reduces neuropathic pain, an analgesic benefit that is largely lacking in opioid monotherapy and other analgesics.

ADV502 was evaluated in oral, subcutaneous, and intraperitoneal administration of animal models of chronic neuropathic pain in osteoarthritis and sciatic nerve pain. ADV502 was shown to be effective and controlled pain in a dose-dependent fashion [13]. Further study showed that ADV502 was associated with less tolerance than oxycodone or morphine at 22 days, fewer and less severe withdrawal symptoms versus oxycodone, and low rates of self-administration compared to cocaine [2]. The bifunctional compound may have an opioid-sparing effect and be associated with fewer of the usual side effects associated with opioid monotherapy, including respiratory depression.

The potential of σ-receptor analogs to manage pain syndromes and neurodegenerative conditions

Pain medicine today is confronting major challenges. The analgesic armamentarium has not been refreshed in many years. Compared to fields such as oncology or cardiovascular medicine, there has been a dearth of innovation in pain medicine. The old analgesic agents have known risks that in some cases outweigh their potential benefits. The opioid crisis has caused many physicians in the United States to refer their pain patients to the very small number of pain specialists to manage even straightforward cases.

Uncontrolled pain not only causes undue suffering but also unleashes a vicious cycle where pain leads to avoidance behaviors which, in turn, decrease mobility, and diminish function and self-efficacy, all of which lead to more pain and start the cycle anew. Pain is a biopsychosocial phenomenon that can diminish the human being; it is a negative yet holistic experience. Some chronic pain patients require care from family members or others; these caregivers can be severely and adversely impacted by the chronic pain syndromes of those in their care, but are rarely considered in discussions about the many costs and consequences of untreated pain.

A fundamental challenge in treating pain is that pain patients are a very heterogeneous population. A cholesterol-lowering drug can effectively treat just the majority of patients with high cholesterol, but pain does not respond as readily to this one-drug-suits-all approach. Pain syndromes are often mixed and require multi-mechanistic solutions which may be complicated and not reimbursable. Factor in nonpharmacological options in a multidisciplinary pain care model, and pain care become prohibitively expensive. Even with the best efforts, many chronic pain patients still experience a certain amount of pain. This is likely the reason that in the management of severe pain syndromes, clinicians always seem to end up at oral opioid monotherapy.

Current research involving σ-receptors has the potential to introduce new drug classes and new therapeutic options for analgesia as well as other indications. There are already a number of σ1 and σ2 receptor agonists and antagonists in various stages of development or commercialization [2]. Many of these σ-receptor agents are gaining attention for their possible benefits in neurodegenerative disorders, psychiatric conditions, and even rare diseases, such as pediatric Rett syndrome, but pain is one of the most important potential indications for these drugs [14]. The σ-receptors act as ligand-activated chaperones to promote protein folding integrity via inter-organelle signaling pathways not yet fully elucidated [15]. They also transport cholesterol and maintain cholesterol homeostasis by aggregating apolipoproteins, monomers, and digomers and modulating oxidative phosphorylation [16]. To accomplish these many tasks, the σ-receptors are known to interact with > 100 proteins. Finally, the σ-receptors play a role in synaptic signaling pathways by modulating ion channels, regulating calcium homeostasis, and controlling the homeostasis of reactive oxygen species [17, 18]. For these tasks, the receptors activate intercellular mitochondrial transfers [18].

Chaperone proteins bind to misfolded proteins in such a way that the conformational errors introduced by misfolding can be corrected or destroyed [8, 9, 19]. A number of these compounds are currently in the pipeline (see Table 2).

**Table 2:** The pipeline of σ-receptor compounds currently under investigation. Some of their potential indications are conditions for which no current first-line treatment is known. Both early and late-stage Alzheimer’s disease are potential disease targets but represent different pharmacological needs. AD, Alzheimer’s disease; PD, Parkinson disease; ped, pediatric

Candidate	Target	Disease(s)	Status
ADV-462	σ-2 antagonist	Early AD Down Syndrome	IND meeting to enter Phase 1
ADV-127	σ-1 agonist	Fragile X PD	Preclinical
ADV-368	σ-1 agonist + σ-2 antagonist	Huntington’s disease Late AD	Preclinical
ADV-422	α-2 δ-1 selective	Ped epilepsy	Preclinical
ADV-502	σ-1 antagonist + µ-opioid receptor partial agonist	Neuropathic pain Cancer pain	Phase 1
ADV-301	σ-2 agonist	Cancer Autoimmune diseases Pain	Entering preclinical
ADV-209	σ-2 agonist + σ-1 antagonist	Cancer Autoimmune diseases Pain	Entering preclinical

The mechanism of action of the neuroprotective agent and candidate drug ADV462 is the prevention and binding of Aβ oligomers and their subsequent removal to the cerebral spinal fluid [20]. This improves cognition. Another candidate drug, CT1812 (Cognition Therapeutics, Purchase, NY, US) is a σ2 antagonist for treating mild to moderate early Alzheimer’s disease [21]. Preclinical studies using microelectrode arrays have assessed the neurotoxicity of amyloid-β1-42 oligomers in CT1812. Unlike monomers, oligomers significantly reduce the network spike rate on the microelectrode array, a common metric for drug activity [22]. In preclinical studies, ADV462 was shown to regulate both synaptic transmission and neuronal plasticity in the synaptic transmissions impaired by Aβ. This action allowed the spike rate to recover, that is, for drug activity to resume. The combination of these agents appears to significantly enhance the effects of CT1812 without adverse effects.

Fragile X syndrome is a genetic neurodevelopmental condition associated with intellectual impairment and autism [23]. It occurs due to an unstable CGG trinucleotide expansion within the Fragile X Mental Retardation 1 (FMR1) gene [24]. This produces a hypermethylated region in the gene promoter, resulting in gene silence and reduced expression of a protein involved in synaptic plasticity and maturation, leading to the condition known as Fragile X Mental Retardation 1 [23]. It has some similarities with Parkinson’s disease, a neurodegenerative disorder associated with older age and involving a lack of dopamine. The underlying conditions of Parkinson's disease include mitochondrial disruption, oxidative stress, an aggregation of proteins, disordered autophagy, and chronic neuroinflammation [25]. Both Fragile X syndrome and Parkinson’s disease are caused by neuronal damage in the brain and may manifest as tremors, movement disorders, and cognitive deficits [26]. Anavex®2-73 (Anavex Life Sciences, New York, NY, US) is a nonselective σ1 agonist in phase 2 clinical trials for the treatment of Fragile X syndrome. The activation of σ1 receptors enhances neuronal plasticity and synaptic connection, activates brain-derived neurotrophic factor (BDNF)-related peripheral signaling lymphocyte pathways, and restores proteostasis.

A novel first-in-class bispecific σ2 antagonist combined with a σ1 agonist has entered phase 3 clinical studies for the indication of Huntington’s disease. Huntington’s disease is a neurodegenerative disorder caused by a CAG trinucleotide repeat expansion in the Huntington gene, resulting in cognitive impairment, motor deficits, and potential psychiatric disorders [27]. It has an adverse effect on proteostasis, transcription, and mitochondrial function, which can result in potentially fatal toxicity. There is no cure or even disease-modifying treatment [28]. A σ1 agonist known as pridopidine is being sponsored by Prilenia Therapeutics. The activation of σ1 helps to clear accumulated toxic proteins, restore energy production, diminish cellular stress, reduce inflammation and, in so doing, improve cognitive and motor function. While this is promising, the novel candidate ADV368 is a combination agent of an σ1 agonist with a σ2 antagonist. The σ1 agonist prevents oligomer and protein aggregation, enhancing neurogenesis and allowing mature neurons to survive. The σ2 receptor antagonist works to remedy aberrant cellular processes, such as autophagy, cholesterol synthesis, and the trafficking of various proteins. This combination product would be a first-in-class bispecific agent.

There are significant, urgent, and unmet medical needs for neuropathic pain in cancer patients [29]. While there are agents for neuropathic cancer pain, such as opioids used together with gabapentinoids or duloxetine, the analgesic benefit is not profound. Drugs that address the σ family of receptors may be particularly useful for neuropathic pain syndromes.

When a peripheral nerve sustains an injury, a dense expression of σ1 receptors occurs. These σ1 receptors are intended to modulate the nociceptive action of the peripheral nervous system, but may in fact drive neuropathic pain instead [30]. Painful peripheral neuropathy in obesity is an emerging condition that has not been thoroughly studied [31], but σ1 receptors may promote N-methyl-D-aspartate receptor (NMDAR) expression in the spine that can mediate the condition. In a murine study, σ1 blockade prevented peripheral diabetic neuropathy [32]. Glaucoma is a form of optic neuropathy that shares some pathologies with Alzheimer's disease and Parkinson’s disease; σ1 may confer neuroprotection against glaucoma [33].

A paradoxical finding in sigma research has been that agonism of the σ1 receptor promotes both neuroprotection and neurodegeneration. This has been explained that when disease advances, σ1 receptors become increasingly dysfunctional and allow neuronal damage to occur; however, when σ1 receptor activity can be promoted and enhanced, neuroprotection occurs [34].

A novel agent in development for the indication of neuropathic cancer pain is ADV502, a high-affinity σ1 receptor antagonist and a partial agonist of the µ-opioid receptor. In terms of efficacy, ADV502 can be fairly compared to strong opioids but may actually be more effective in reducing neuropathic pain. Its σ1 component reduces the recruitment of β-arrestin which means there is a low tolerance decrement in terms of analgesic benefit [35, 36]. It also has fewer of the well-known and potentially treatment-limiting opioid side effects, such as gastrointestinal problems and symptoms related to the central nervous system. It is also safer during naloxone-induced withdrawal. In fact, the reduced abuse potential of this agent makes it very intriguing. It is known to result in less physical dependence, lower “likeability,” and it has fewer and less severe withdrawal symptoms than conventional opioid monotherapy, which suggests - but does not prove - that it may have a lower abuse potential than opioid monotherapy. This novel drug candidate is an oral product that may treat neuropathic as well as nociceptive pain with better tolerability, lower risk of respiratory depression, and reduced withdrawal symptoms compared to strong opioids alone [2].

The potential of ADV502 for opioid use disorder

There have been few innovations in medications for medication-assisted treatment of opioid use disorder since buprenorphine and methadone. Methadone has been used since 1947 for the treatment of opioid use disorder and many clinical studies support its use [37]. Patients with opioid use disorder treated with methadone had 33% fewer opioid-positive drug tests. Moreover, people taking methadone for opioid use disorder were more than four times as likely to stay in a rehabilitation program than controls [38]. Even when methadone is provided without adjunctive counseling or support services, it still improves outcomes. Regardless of the counseling component, long-term outcomes, defined as those of more than six months, are better for methadone patients than those not taking methadone [38]. Despite these benefits, there are some drawbacks to methadone maintenance therapy. Methadone is a full opioid agonist, so if it is misused or overdosed, it can result in opioid-induced respiratory depression. Its pharmacokinetics are variable and there is a risk of prolonged QTc intervals, which, in turn, may induce potentially dangerous arrhythmias [39, 40].

The newer agent, buprenorphine, was approved in 2002 and is available for opioid use disorder as a monotherapeutic agent or in combination with naloxone, an opioid receptor antagonist. It is not a short-term solution, because those tapered off buprenorphine have a higher rate of relapse than patients maintained on buprenorphine for a longer period of time. Those administered doses of at least 16 mg/day of buprenorphine were nearly twice as likely to stay in opioid rehabilitation treatment compared to placebo patients [38]. Patients taking buprenorphine had 14% fewer opioid-positive drug tests [38]. It appears that the effectiveness of buprenorphine for treating opioid use disorder depends on the dose. A limitation of buprenorphine therapy is that there are a limited number of clinicians available to prescribe and dispense the treatment [41]. Costs for buprenorphine are higher than for methadone and may be prohibitive [38]. There is also a degree of confusion about the role of buprenorphine because it is known as both a strong analgesic and as a maintenance drug. There can also be confusion about proper dosing and optimal routes of administration for these two distinct indications.

A Cochrane review and meta-analysis compared buprenorphine, methadone, and placebo for treating people with opioid use disorder and found no significant differences in opioid-positive drug screens or self-reported heroin use when methadone or buprenorphine was administered at medium to high doses, but methadone was superior to buprenorphine in terms of treatment retention [42].

As an µ-opioid receptor antagonist, naltrexone is a different type of agent in opioid rehabilitation. It was originally approved in 2010 as an oral pill to be taken once daily, but lack of adherence has limited our data in terms of measuring its effectiveness. Nowadays, an injectable extended-release naltrexone formulation is used monthly, which improved adherence by eliminating the need for daily doses. In a clinical study of opioid use disorder patients, those using extended-release naltrexone had 90% opioid-abstinent weeks versus 35% in the placebo group. Naltrexone patients were also more likely than placebo patients to be treatment adherent (58% versus 42%). Naltrexone reduces drug cravings, but its effectiveness is greater among highly motivated patients [38].

These current options are in widespread use, albeit with limitations. Their short-term efficacy appears to be good but better data are needed to confirm long-term efficacy, which may be in part dependent on the degree of support the patient receives. The role of counseling or support the patient receives likely plays a role in treatment success but has not been well studied. Patient support can range from one-on-one psychiatric sessions to online patient support groups, group therapy led by peers, or sessions with licensed or peer counselors. Not all patients receive counseling, but when counseling is offered, the many different types and styles of psychological support make comparisons difficult. Safety issues are particularly important with methadone. Cost and availability limit the use of buprenorphine along with the fact that buprenorphine efficacy depends on proper dosing, but dosing has a high degree of interpatient variability. Finally, none of these agents were originally developed or designed as drugs to treat opioid use disorder [38].

**Figure 1:**
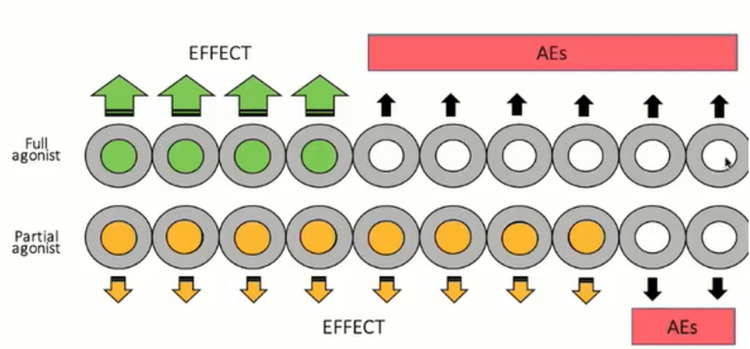
A full agonist can produce effects without occupying all receptors, but these unoccupied receptors may be associated with adverse effects. A partial agonist is associated with few, if any, unoccupied receptors and fewer adverse effects. Figure produced by Robert B. Raffa, PhD., from his presentation and used with permission.

Methadone is distinct from buprenorphine, in that methadone is a full agonist at the µ-opioid receptors, while buprenorphine is a partial agonist at the µ-opioid receptor and has putative effects on δ- and κ-opioid receptors. Methadone has a complex pharmacology and long half-life [43]. It is important to understand that although buprenorphine is a partial agonist at the µ-opioid receptor, it has full - not partial - effect as an analgesic agent [44]. A partial agonist does not reduce the effect to zero or act like an antagonist. Buprenorphine also exerts activity at the nociception/ orphanin FQ (NOP) receptors [45]. The benefit of a partial agonist is that it must occupy many receptors to achieve its result, while a full agonist need only occupy a subset of receptors to have the same effect. This means that for a full agonist, there are many “spare receptors” which can cause adverse effects (see Figure 1).

**Figure 2:**
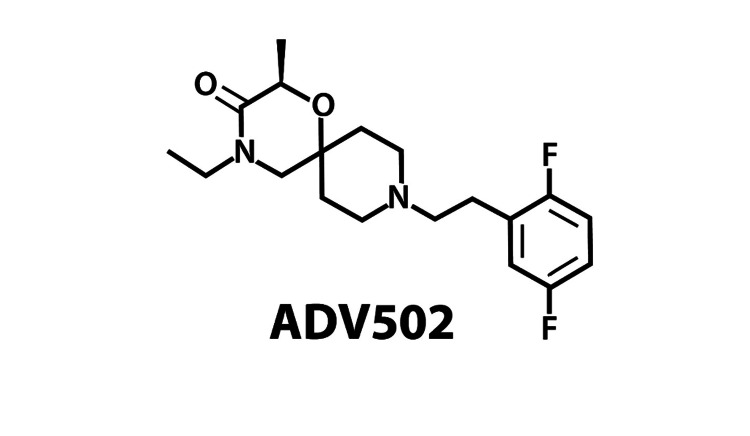
The drug candidate ADV502 has 118±6 Ki, nanomole affinity for the σ-1 receptor, and 750 to >1000 (glycoprotein) Ki, nanomole for the σ-2 receptor. These indicate selectivity. It is also balanced as the drug as µ-receptor affinity of 64±5 Ki, nanomole. This in vitro balance was observed in images of rat brains as well, showing good in vivo balance. This figure courtesy of Advantx Pharmaceuticals.

This novel combination product of an opioid analgesic plus a σ1 antagonist may be effective for the treatment of opioid use disorder. A study of dezocine suggests that a combination drug that acts as a σ-ligand might reduce opioid use disorder using a murine model of morphine dependence [46]. A strategy would be to develop a product that was a partial opioid agonist (similar to buprenorphine), a biased ligand (similar to oliceridine), and a selective σ-1 antagonist. This combination should be made in such a way that there is a balanced contribution to both the µ-opioid receptor and the σ1 receptor. This type of bifunctional product has been the subject of drug discovery efforts [10, 11, 13]. A novel σ1 selective chemical entity has been introduced as ADV502 (see Figure 2).

ADV502 has been classified as a partial µ-opioid receptor and a highly biased ligand of the σ receptor. In a rodent study of self-administration, rats preferred ADV502 over water if it only took a single lever press to obtain it [2]. However, if work was increased so that rats had to press a lever three times to get ADV502 or one time to obtain water, they selected water. In a comparative test, rats preferred cocaine over water even if it took multiple presses to obtain the cocaine [2].

ADV502 shows tremendous promise as a potential new agent to address opioid use disorder and it may be a welcome addition to our limited armamentarium of agents for opioid rehabilitation.

## Conclusions

After many years of doldrums in pain pharmacology innovation, new compounds targeting sigma receptors hold promise not just for pain care but for the treatment of many diverse conditions, ranging from Alzheimer’s disease and other neurodegenerative disorders to cancer. These recently discovered receptors are ligand-activated protein chaperones, the mechanisms of which are still being elucidated. Agents that act as agonists or antagonists of sigma receptors are in the pipeline of drug development. Some agents are first-in-class drugs and many hold promise for conditions, such as neuropathic pain, neurodegenerative diseases, and Fragile X syndrome, which are challenging to treat pharmacologically. Among these new agents is ADV502, a novel bifunctional compound that may treat pain or also be used for opioid rehabilitation therapy. With greater understanding of the role of sigma receptors in the body and the ability to target these receptors with specific agents, important new treatment options for long-standing irreversible conditions, such as Alzheimer's disease, may soon be viable.

## References

[1] Martin WR, Eades CG, Thompson JA, Huppler RE, Gilbert PE (1976). The effects of morphine- and nalorphine- like drugs in the nondependent and morphine-dependent chronic spinal dog. J Pharmacol Exp Ther.

[2] Pergolizzi J, Varrassi G, Coleman M, Breve F, Christo DK, Christo PJ, Moussa C (2023). The sigma enigma: A narrative review of sigma receptors. Cureus.

[3] Hanner M, Moebius FF, Flandorfer A, Knaus HG, Striessnig J, Kempner E, Glossmann H (1996). Purification, molecular cloning, and expression of the mammalian sigma1-binding site. Proc Natl Acad Sci U S A.

[4] Schmidt HR, Zheng S, Gurpinar E, Koehl A, Manglik A, Kruse AC (2016). Crystal structure of the human σ1 receptor. Nature.

[5] Alon A, Schmidt H, Zheng S, Kruse AC (2017). Structural perspectives on sigma-1 receptor function. Adv Exp Med Biol.

[6] Alon A, Lyu J, Braz JM (2021). Structures of the σ(2) receptor enable docking for bioactive ligand discovery. Nature.

[7] Yasui Y, Su TP (2016). Potential molecular mechanisms on the role of the sigma-1 receptor in the action of cocaine and methamphetamine. J Drug Alcohol Res.

[8] Wang M, Kaufman RJ (2016). Protein misfolding in the endoplasmic reticulum as a conduit to human disease. Nature.

[9] Moreno-Gonzalez I, Soto C (2011). Misfolded protein aggregates: mechanisms, structures and potential for disease transmission. Semin Cell Dev Biol.

[10] Zhuang T, Xiong J, Hao S (2021). Bifunctional μ opioid and σ(1) receptor ligands as novel analgesics with reduced side effects. Eur J Med Chem.

[11] García M, Virgili M, Alonso M (2020). 4-Aryl-1-oxa-4,9-diazaspiro[5.5]undecane derivatives as dual μ-opioid receptor agonists and σ(1) receptor antagonists for the treatment of pain. J Med Chem.

[12] Ayet E, Yeste S, Reinoso RF, Pretel MJ, Balada A, Serafini MT (2021). Preliminary in vitro approach to evaluate the drug-drug interaction potential of EST73502, a dual µ-opioid receptor partial agonist and σ1 receptor antagonist. Xenobiotica.

[13] García M, Virgili M, Alonso M (2020). Discovery of EST73502, a dual μ-opioid receptor agonist and σ(1) receptor antagonist clinical candidate for the treatment of pain. J Med Chem.

[14] Abate C, Niso M, Berardi F (2018). Sigma-2 receptor: past, present and perspectives on multiple therapeutic exploitations. Future Med Chem.

[15] Su TP, Hayashi T, Maurice T, Buch S, Ruoho AE (2010). The sigma-1 receptor chaperone as an inter-organelle signaling modulator. Trends Pharmacol Sci.

[16] Hayashi T, Su TP (2003). Sigma-1 receptors (sigma(1) binding sites) form raft-like microdomains and target lipid droplets on the endoplasmic reticulum: roles in endoplasmic reticulum lipid compartmentalization and export. J Pharmacol Exp Ther.

[17] Meunier J, Hayashi T (2010). Sigma-1 receptors regulate Bcl-2 expression by reactive oxygen species-dependent transcriptional regulation of nuclear factor kappaB. J Pharmacol Exp Ther.

[18] Weng TY, Tsai SA, Su TP (2017). Roles of sigma-1 receptors on mitochondrial functions relevant to neurodegenerative diseases. J Biomed Sci.

[19] Gregersen N, Bross P, Vang S, Christensen JH (2006). Protein misfolding and human disease. Annu Rev Genomics Hum Genet.

[20] LaBarbera KM, Sheline YI, Izzo NJ (2023). A phase 1b randomized clinical trial of CT1812 to measure Aβ oligomer displacement in Alzheimer's disease using an indwelling CSF catheter. Transl Neurodegener.

[21] Rishton GM, Look GC, Ni ZJ (2021). Discovery of investigational drug CT1812, an antagonist of the sigma-2 receptor complex for Alzheimer’s disease. ACS Med Chem Lett.

[22] Johnstone AF, Gross GW, Weiss DG, Schroeder OH, Gramowski A, Shafer TJ (2010). Microelectrode arrays: a physiologically based neurotoxicity testing platform for the 21st century. Neurotoxicology.

[23] Saldarriaga W, Tassone F, González-Teshima LY, Forero-Forero JV, Ayala-Zapata S, Hagerman R (2014). Fragile X syndrome. Colomb Med (Cali).

[24] Terracciano A, Chiurazzi P, Neri G (2005). Fragile X syndrome. Am J Med Genet C Semin Med Genet.

[25] Simon DK, Tanner CM, Brundin P (2020). Parkinson disease epidemiology, pathology, genetics, and pathophysiology. Clin Geriatr Med.

[26] Kalin NH (2022). Neurodevelopmental and neurodegenerative illnesses: Autism, Fragile X syndrome, Parkinson's disease, and dementia. Am J Psychiatry.

[27] Ghosh R, Tabrizi SJ (2018). Clinical features of Huntington's disease. Adv Exp Med Biol.

[28] McColgan P, Tabrizi SJ (2018). Huntington's disease: a clinical review. Eur J Neurol.

[29] Varrassi G, De Conno F, Orsi L, Puntillo F, Sotgiu G, Zeppetella J, Zucco F (2020). Cancer pain management: An Italian Delphi survey from the Rational Use of Analgesics (RUA) Group. J Pain Res.

[30] Puente B, Nadal X, Portillo-Salido E (2009). Sigma-1 receptors regulate activity-induced spinal sensitization and neuropathic pain after peripheral nerve injury. Pain.

[31] O'Brien PD, Hinder LM, Callaghan BC, Feldman EL (2017). Neurological consequences of obesity. Lancet Neurol.

[32] Paniagua N, Girón R, Goicoechea C, López-Miranda V, Vela JM, Merlos M, Martín Fontelles MI (2017). Blockade of sigma 1 receptors alleviates sensory signs of diabetic neuropathy in rats. Eur J Pain.

[33] Xu Z, Lei Y, Qin H, Zhang S, Li P, Yao K (2022). Sigma-1 receptor in retina: neuroprotective effects and potential mechanisms. Int J Mol Sci.

[34] Nguyen L, Lucke-Wold BP, Mookerjee S, Kaushal N, Matsumoto RR (2017). Sigma-1 receptors and neurodegenerative diseases: Towards a hypothesis of sigma-1 receptors as amplifiers of neurodegeneration and neuroprotection. Adv Exp Med Biol.

[35] van Gastel J, Hendrickx JO, Leysen H, Santos-Otte P, Luttrell LM, Martin B, Maudsley S (2018). β-Arrestin based receptor signaling paradigms: Potential therapeutic targets for complex age-related disorders. Front Pharmacol.

[36] Gress K, Charipova K, Jung JW (2020). A comprehensive review of partial opioid agonists for the treatment of chronic pain. Best Pract Res Clin Anaesthesiol.

[37] Joudrey PJ, Edelman EJ, Wang EA (2020). Methadone for opioid use disorder-Decades of effectiveness but still miles away in the US. JAMA Psychiatry.

[38] (2023). National Institute on Drug Abuse. How effective are medications to treat opioid use disorder?. https://nida.nih.gov/publications/research-reports/medications-to-treat-opioid-addiction/efficacy-medications-opioid-use-disorder.

[39] Ehret GB, Voide C, Gex-Fabry M (2006). Drug-induced long QT syndrome in injection drug users receiving methadone: high frequency in hospitalized patients and risk factors. Arch Intern Med.

[40] Chou R, Weimer MB, Dana T (2014). Methadone overdose and cardiac arrhythmia potential: findings from a review of the evidence for an American Pain Society and College on Problems of Drug Dependence clinical practice guideline. J Pain.

[41] Pergolizzi J, LeQuang JA, Breve F (2021). The end of the X-waiver: not a moment too soon!. Cureus.

[42] Mattick RP, Breen C, Kimber J, Davoli M (2014). Buprenorphine maintenance versus placebo or methadone maintenance for opioid dependence. Cochrane Database Syst Rev.

[43] Kreutzwiser D, Tawfic QA (2020). Methadone for pain management: A pharmacotherapeutic review. CNS Drugs.

[44] Raffa RB, Haidery M, Huang HM (2014). The clinical analgesic efficacy of buprenorphine. J Clin Pharm Ther.

[45] Pergolizzi J, Aloisi AM, Dahan A (2010). Current knowledge of buprenorphine and its unique pharmacological profile. Pain Pract.

[46] Wu FX, Babazada H, Gao H (2019). Dezocine alleviates morphine-induced dependence in rats. Anesth Analg.

